# Novel *XIAP* mutation causing enhanced spontaneous apoptosis and disturbed NOD2 signalling in a patient with atypical adult-onset Crohn’s disease

**DOI:** 10.1038/s41419-020-2652-4

**Published:** 2020-06-08

**Authors:** Zuzana Parackova, Tomas Milota, Petra Vrabcova, Jitka Smetanova, Michael Svaton, Tomas Freiberger, Veronika Kanderova, Anna Sediva

**Affiliations:** 1 Department of Immunology, 2nd Faculty of Medicine Charles University, University Hospital in Motol, V Uvalu 84, Prague, Czech Republic; 2 CLIP—Childhood Leukaemia Investigation Prague, Department of Paediatric Haematology and Oncology, 2nd Faculty of Medicine, Charles University and University Hospital Motol, Prague, Czech Republic; 3 Molecular Genetics Laboratory, Center of Cardiovascular Surgery and Transplantation, Brno, Czech Republic; 4 Faculty of Medicine, Masaryk University, Brno, Czech Republic

**Keywords:** Immune cell death, Crohn's disease

## Abstract

X-linked inhibitor of apoptosis (XIAP) is the most potent human inhibitor of apoptosis, and is also involved in NOD2-dependent NFκB and MAPK signalling cascade activation. The absence or defective function of XIAP leads to the development of a rare and severe primary immunodeficiency known as X-linked lymphoproliferative syndrome type 2 (XLP-2), which is characterized by a triad of clinical manifestations, including a high incidence of haemophagocytic lymphohistiocytosis (HLH), lymphoproliferation and inflammatory bowel disease (IBD), usually with very early onset. Here, we present a novel *XIAP* mutation identified in a patient with atypical adult-onset IBD complicated by relapsing HLH, splenomegaly and sarcoid-like disease. The c.266delA mutation in the *XIAP* gene creates a premature stop codon, and causes a severe reduction in XIAP protein expression. The mutation is also associated with impaired spontaneous and staurosporine- and PMA-induced apoptosis accompanied by significantly increased expression of pro-apoptotic genes. We also confirmed the negative impact of this particular *XIAP* mutation on NOD2-dependent NFκB and MAPK activation, while NOD2-independent activation was found to be unaffected. Moreover, we assume that the mutation has an impact on the overproduction of IL-12 and IFNγ, the shift towards the Th1 immune response and increased numbers of central memory and effector memory CD4+ and CD8+ T cells. All these changes contribute to immune dysregulation and the clinical manifestation of XLP-2.

## Introduction

X-linked inhibitor of apoptosis (XIAP) or baculoviral IAP repeat-containing protein 4 (BIRC4), localized on the X chromosome, is a part of human IAP family. The protein consists of three different domains: (1) three baculoviral IAP repeat (BIR) domains, which are characteristic of all IAPs, (2) UBA domains that allow binding to ubiquitin and (3) a zinc-binding domain C-terminal RING finger domain, which is associated with E3 ubiquitin ligase activity^1^.

One of the major roles of XIAP is the prevention of apoptotic cell death, which is achieved by binding and inhibiting the activity of caspases 3, 7 and 9^2^. In addition to its anti-apoptotic functions, XIAP is also involved in other signalling pathways and cellular responses, mostly because of the ubiquitylation activity through its RING domain^3,4^. XIAP is involved in intracellular pattern-recognition receptor signalling that senses peptidoglycan products, NOD1 and 2^5^, leading to NFκB and mitogen-activated protein kinase (MAPK) cascade activation^6–8^. In mouse and human models, the absence of XIAP leads to defective secretion of proinflammatory cytokines after stimulation with NOD ligands^9,10^. Interestingly, NOD2 was the first identified susceptibility gene for Crohn’s disease (CD), a typical condition associated with XIAP deficiency^11^.

XIAP deficiency is a rare primary immunodeficiency, also known as X-linked lymphoproliferative syndrome type 2 (XLP-2), caused by mutations in the *XIAP (BIRC4)* gene. The estimated incidence is 1–2 cases per million of live-born children. Nevertheless, the real prevalence seems to be higher as the diagnosis of XIAP deficiency may be overlooked or misclassified. Current assessments suggest that up to 4% of early-onset IBD may represent XIAP-deficient patients^12^.

Disease onset usually manifests in the first few years of life, and is characterized by a key triad of clinical symptoms consistent with a high incidence of haemophagocytic lymphohistiocytosis (HLH), often triggered by Epstein–Barr (EBV) infections, and characterized by splenomegaly and inflammatory bowel disease (IBD), particularly with features of CD^13^. HLH is a life-threatening condition characterized by hyperinflammation, in which activated T lymphocytes and macrophages accumulate in organs, and produce and induce massive production of proinflammatory cytokines, particularly IFNγ^14^, resulting in tissue damage and multiorgan failure that typically affects the liver and bone marrow^15^. IBD in XIAP-deficient patients usually presents with very early onset^16^; however, adult onset has also been described^17^, and is characterized by a complicated course, necessity of extensive surgical procedures and unresponsiveness to standard treatment, including biological treatment. These patients have also significantly increased mortality rate, dying within a few years upon manifestation or diagnosis of IBD^18^. In comparison with XLP-1, hypogammaglobulinaemia may accompany XIAP deficiency; however, it is less frequent. Moreover, no lymphoma has been reported, which approximately 30% of XLP-1 patients develop. On the other hand, XLP-1 does not present with higher risk of IBD^19^. Currently, haematopoietic stem cell transplantation is the only causal therapy of XLP-2, although attempts to develop targeted gene therapy seem to be promising^20^.

Here, we report a novel XLP-2-causing mutation in the XIAP BIR1 domain, leading to a premature stop codon and a loss of protein expression, which results in impaired lymphocyte apoptosis and NOD2-dependent signalling with clinical manifestations that include a complicated course of IBD, unresponsiveness to standard treatment, including biologics (infliximab and vedolizumab) and relapsing HLH.

## Results

### Case report

**Fig. 1:**
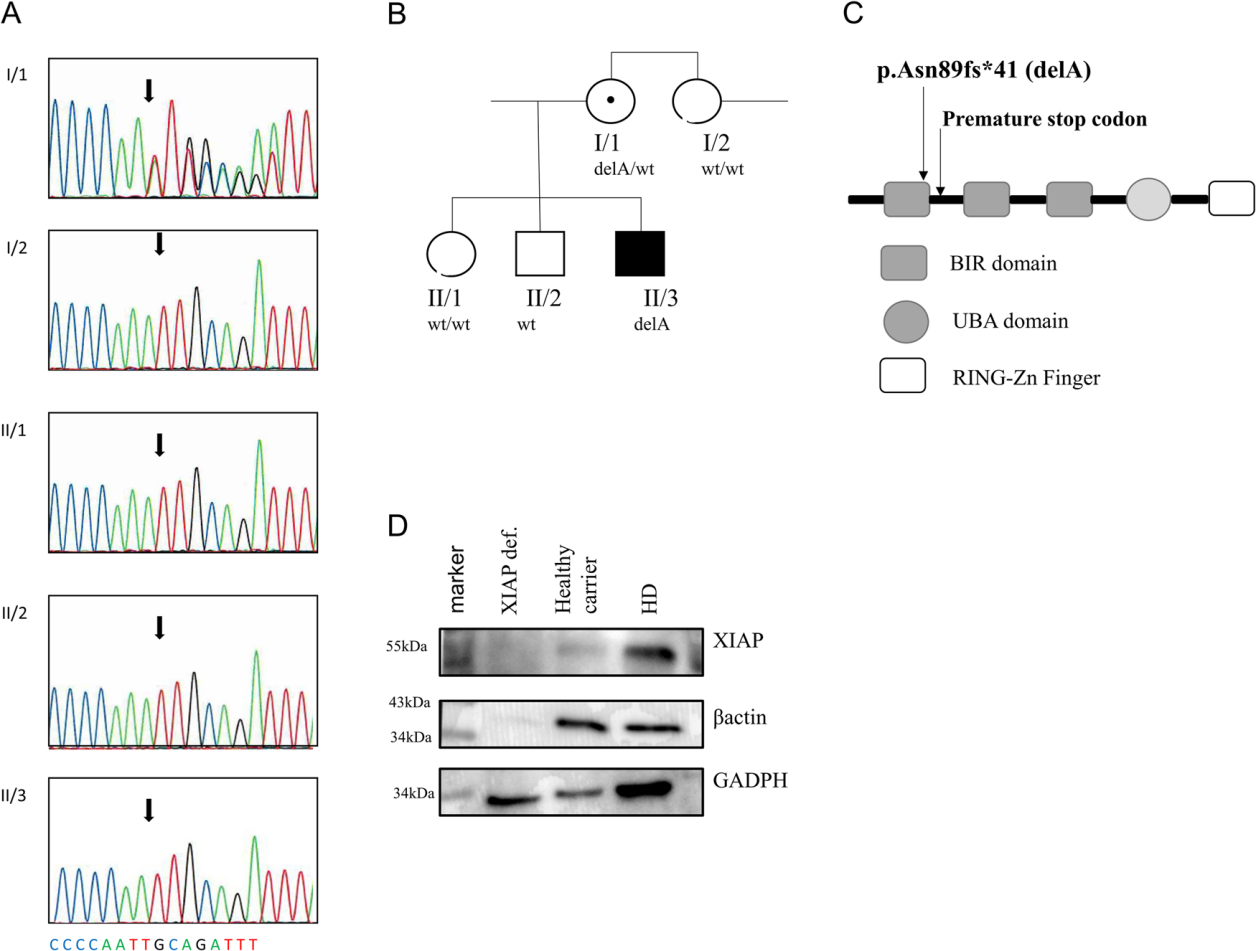
c.266delA mutation. **a** DNA-sequencing chromatogram of the relevant *XIAP* gene regions in the patient (II/3) and his first-degree relatives. The same mutation was detected in a heterozygous form in his mother (I/1), while other relatives carry wild-type (WT) alleles. Arrows show the mutation position. **b** Pedigree of the family showing segregation of the *XIAP* mutation. **c** Protein structure with highlighted position of the mutation. **d** Western blot analysis of XIAP presence in the PBMCs of the patient, his mother and the controls.

A 32-year-old patient was born to non-consanguineous Caucasian parents. The patient presented without any health complications or abnormalities during the prenatal, perinatal and postnatal periods, and was diagnosed at 17 years of age with CD based on the clinical presentation and histological verification, which revealed nonspecific granulation tissue composed of multinucleated giant cells and lymphocytic infiltration in the submucosa of the colon. Complex examination, including ultrasonography of the abdomen, also revealed splenomegaly. Standard therapy with chimeric monoclonal anti-TNFα antibody (infliximab) at a standard dose of 5 mg/kg was initiated. However, the course of the CD was complicated by the development of an intra-abdominal abscess compressing the bladder, which required surgical intervention. Then, the biological therapy was switched to fully human monoclonal anti-TNFα (adalimumab), which successfully led to CD remission. Three years later (at the age of 20), the patient was admitted to the hospital for fever, elevation of inflammatory markers (including C-reactive protein), progressive splenomegaly, anaemia, leukocytopenia and decreased platelet count. Further testing revealed hypertriglyceridaemia, elevated transaminases and increased serum concentrations of ferritin. The results from extensive infectious diagnostic work identified the EBV as a possible trigger. The evaluation of bone marrow biopsy samples confirmed the suspicion of HLH. Thus, according to the Histocyte Society standards, the HLH diagnostic criteria were fulfilled, and adequate therapy started with a high-dose corticosteroid regimen (1000 mg of Solu-Medrol per day) for 3 consecutive days and intravenously administered cyclosporine at a dosage of 2 mg/kg/day, which led to normalization of the blood count values and inflammatory marker, liver transaminase, triglyceride and ferritin levels (Supplementary Table 1 and Table 1). Later, the therapy was switched to peroral corticosteroids and cyclosporin as long-term maintenance therapy. Despite this effort, HLH relapse occurred 4 years later (at the age of 24), and no infection or any other trigger was identified. Moreover, the patient developed mediastinal lymphadenopathy, histologically verified as epithelioid granuloma with images indicative of a sarcoid-like disease. Clinical manifestations and therapy are illustrated in Supplementary Fig. 1A. Suspicions about the primary aetiology arose despite the patient’s age, and genetic testing was indicated. WES was performed because of the broad differential diagnosis of HLH and monogenic causes of CD, and the results revealed a novel c.266delA mutation in the *XIAP* (*BIRC4*) gene. This finding was subsequently confirmed by Sanger sequencing (Fig. 1a). Further genetic counselling with the patient’s family members revealed that the patient’s mother as a healthy carrier and two healthy siblings were without the mutation (Fig. 1b). When we searched the patient′s pedigree, we also identified the mother’s brother as a potentially affected family member who died of severe infection-induced sepsis accompanied by splenomegaly and lymphadenopathy (major symptoms of HLH); however, biological material was not available for genetic testing to confirm the diagnosis or the cause of death.

**Table 1:** Laboratory values of the patient samples.

Immunology	Patient’s values	Referential value
IgG (g/l)	13.00	7.65–13.60
IgG1 (g/l)	7.13	4.9–11.4
IgG2 (g/l)	4.13	1.50–6.40
IgG3 (g/l)	0.316	0.2–1.1
IgG4 (g/l)	0.342	0.08–1.4
IgA (g/l)	2.03	0.91–2.9
IgM (g/l)	↓ 0.38	0.47–1.95
IgE (IU/ml)	↑2.161	0–150
C3 (g/l)	0.98	0.83–2.25
C4 (g/l)	0.22	0.14–0.35
Tetanus (IU/ml)	1.01	0.1
Haemphilus (IU/ml)	9.00	6.00
ANA	neg	–
ANCA	pos (p-ANCA)	–
RF IgG (IU/ml)	4.4	0–22
RF IgA (IU/ml)	2.3	0–22
RF IgM (IU/ml)	2.7	0–22
aTRG (IU/ml)	2.48	0–10
ASCA IgG (IU/ml)	↑ 43.713	0–10
ASCA IgA (IU/ml)	↑ 12.36	0–10

### Novel c.266delA mutation leads to a premature stop codon and loss of function of the XIAP molecule

A novel c.266delA frameshift mutation in the *XIAP* gene of the patient, leading to a premature stop codon after the translation of 41 amino acids (p. Asn89fs*41), was detected by whole-exome sequencing (WES) and confirmed by Sanger sequencing (Fig. 1a). The mother of the patient was confirmed to be a healthy heterozygous carrier (Fig. 1b). The mutation is in the first BIR domain of the protein, as shown in the scheme of the XIAP protein in Fig. 1c. The results from a Western blot analysis showed no XIAP expression in the patient PBMCs and reduced expression of XIAP in the mother’s samples compared with healthy donors (Fig. 1d). We also observed reduced expression of the housekeeping protein β-actin, a finding in agreement with a previously reported role of XIAP in cytoskeleton regulation with reduced β-actin expression^21^. Expression of HSP90 and tubulin, additional housekeeping proteins, was comparable to controls (Supplementary Fig. 1E).

### XIAP LOF mutation results in augmented apoptosis

**Fig. 2:**
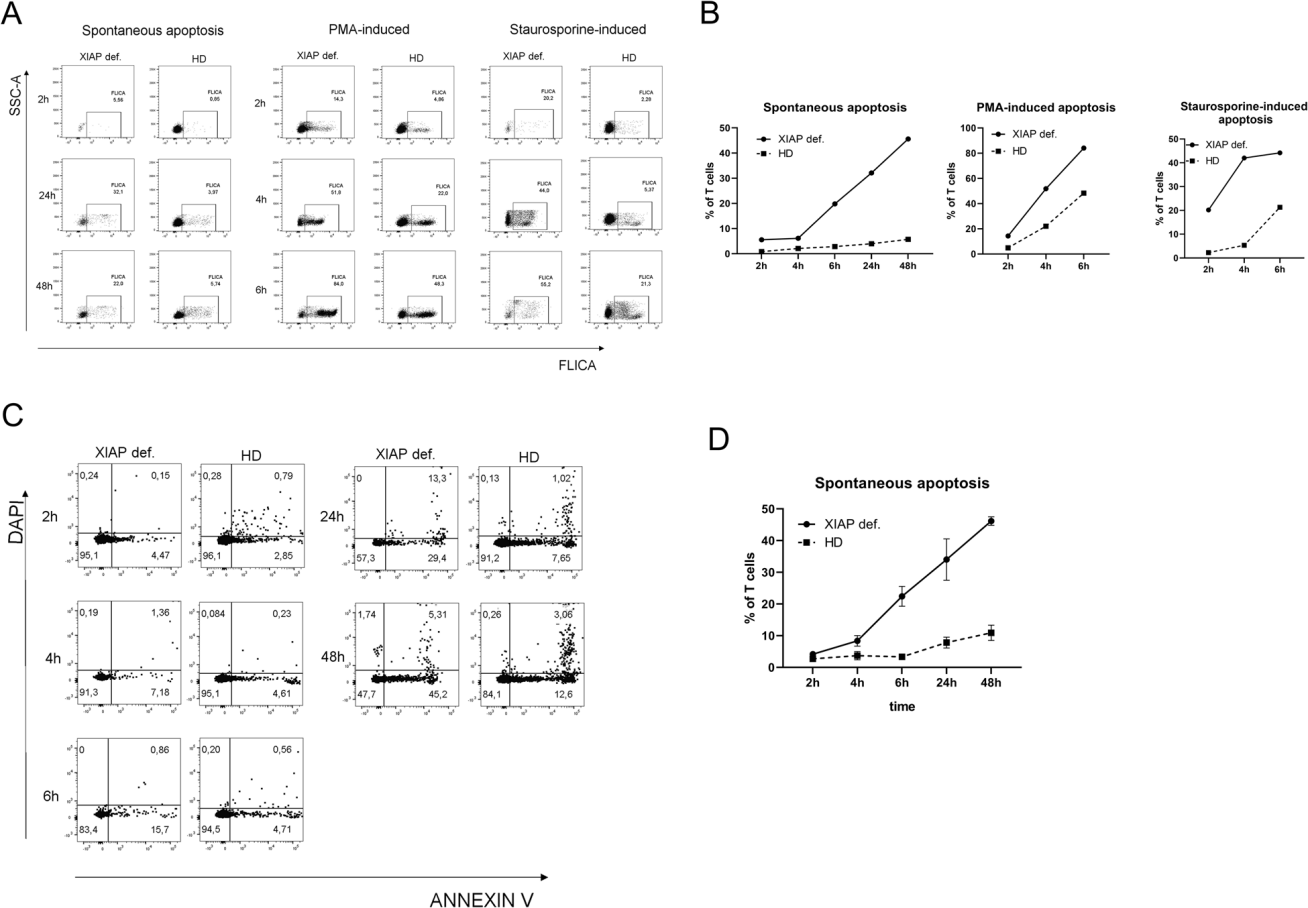
Apoptosis. Patient PBMCs were treated with staurosporine (1 mmol) and 50 ng/ml PMA for 2, 4 and 6 h, or left untreated for an additional 24 and 48 h. The level of spontaneous and induced apoptosis was detected by **a** and **b** FLICA, in which the fluorescein-labelled inhibitor Z-YVAD-fmk is bound to activated caspase-3 and -7 signals as detected by flow cytometry. **c**, **d** The level of spontaneous apoptosis detected by flow cytometry of cells stained with Annexin V and DAPI. Annexin + DAPI cells were considered to be undergoing early apoptosis.

As XIAP is an important molecule in apoptosis regulation, we decided to verify the XIAP LOF by analyzing spontaneous as well as induced apoptosis by staurosporine and PMA. We measured the activation of caspase-3 and -7 with a FAM-FLICA caspase-3,7 assay kit, and noticed elevated numbers of CD3 lymphocytes that were positive for activated caspase-3 and -7 in patient samples. Not only was staurosporine and PMA-induced apoptosis, but also spontaneous apoptosis was markedly enhanced in the patient’s T lymphocytes (Fig. 2a, b). The augmented spontaneous apoptosis was confirmed by Annexin V and DAPI staining, verifying the results of the FLICA experiments (Fig. 2c, d).

**Fig. 3:**
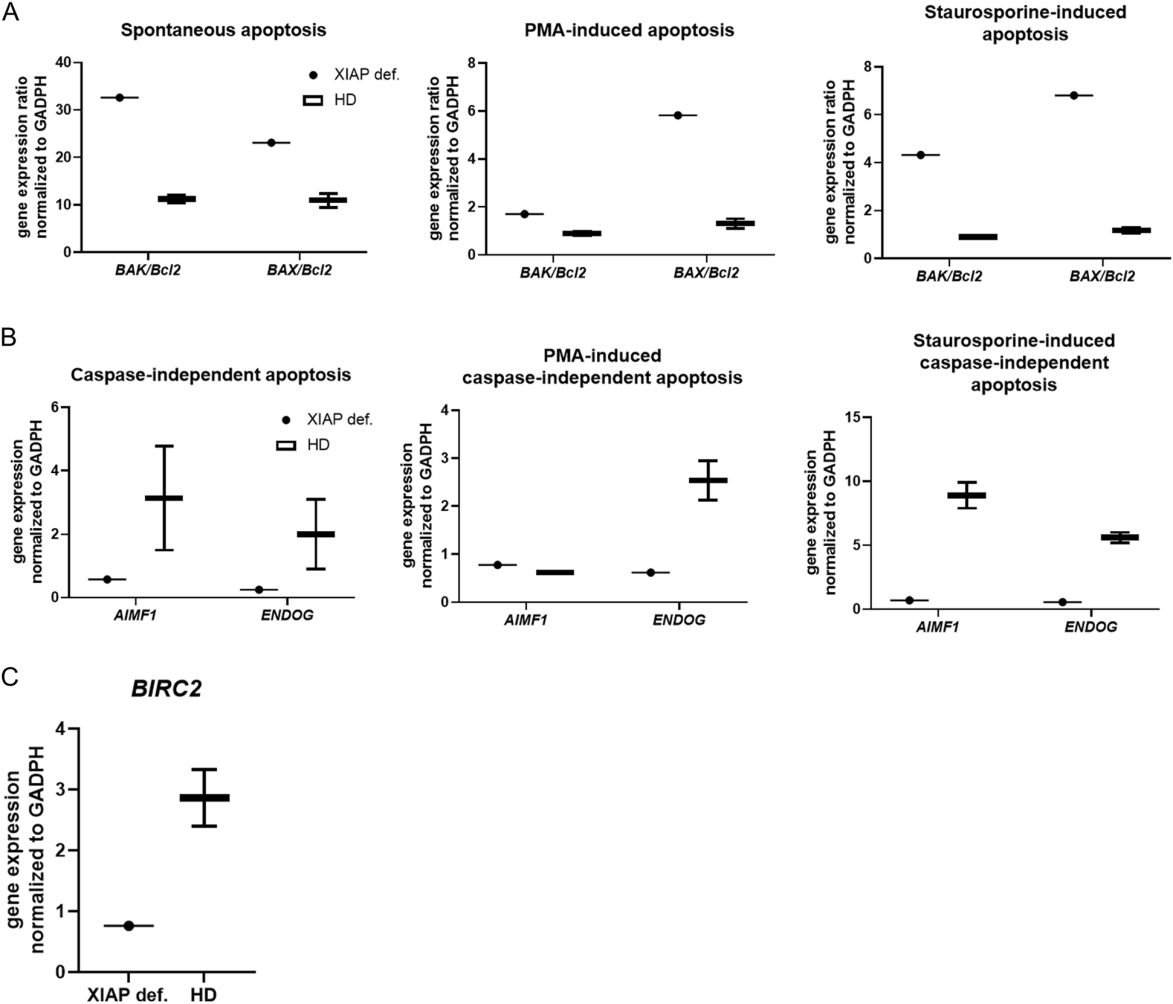
Apoptotic genes’ expression. **a** Ratio of pro- and anti-apoptotic genes *BAK*, *BAX* and *Bcl2* in the patient and control (*n* = 2) PBMCs after 6 h of stimulation with staurosporine (1 mmol) or PMA (50 ng/ml) as detected by RT-PCR. **b** Expression of caspase-independent genes involved in apoptosis, *AIMF1* and *ENDOG*, in the patient and control (*n* = 2) PBMCs after 6 h of stimulation with staurosporine (1 mmol) or PMA (50 ng/ml) as detected by RT-PCR. **c** Expression of the *BIRC2* gene in control (*n* = 2) PBMCs as detected by RT-PCR. Gene expression was normalized to that of *GAPDH*.

In addition, we analyzed the expression of pro-apoptotic (*BAX* and *BAK*) and anti-apoptotic (*Bcl2*) genes. The ratio of *BAK/Bcl2* and *BAX/Bcl2* was highly increased in both induced and spontaneous apoptotic patient cells (Fig. 3a). Interestingly, the genes involved in caspase-independent apoptosis, *ENDOG* and *AIMF1*, were reduced in the samples (Fig. 3b). When a caspase inhibitor Z-VAD-FMK was applied, both patient and control samples displayed reduced apoptosis (Supplementary Fig. 2A, B). These observations suggest an enhanced caspase-dependent apoptosis. To test whether there was a compensatory mechanism critical for defective *XIAP* expression, we analyzed the presence of the *BIRC2* (*cIAP*) gene in patient cells. However, we did not observe enhanced compensatory *cIAP* expression in patient cells compared with healthy controls (Fig. 3c).

### XIAP LOF abrogates NOD2 signalling

**Fig. 4:**
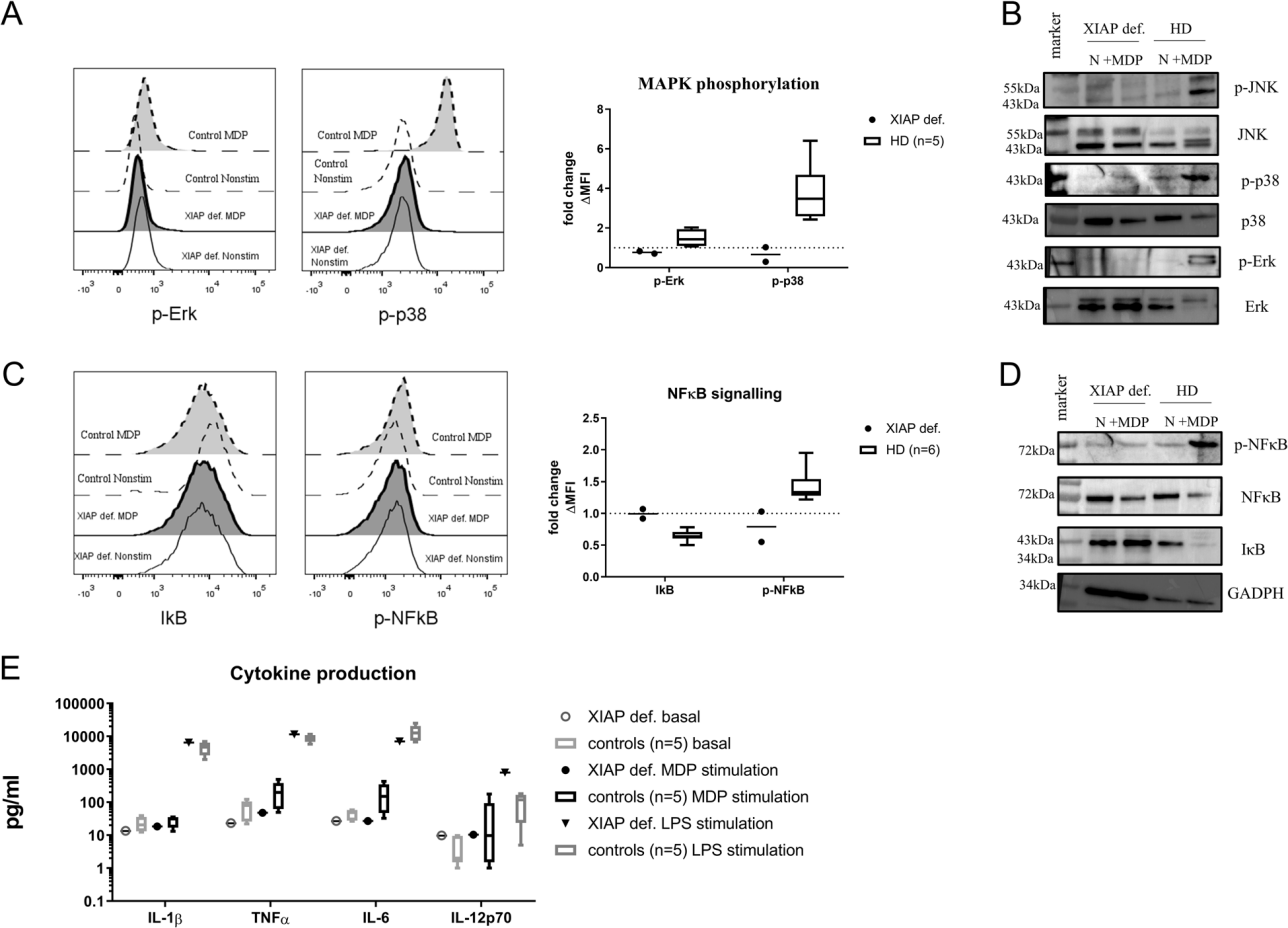
NOD2 signalling. Patient and control (*n* = 5) peripheral blood was stimulated with MDP (10 µg/ml) for 20 min, and phosphorylation of **a** MAP kinases were detected by phospho-flow cytometry **b** and by Western blot. NFκB signalling, expressed as IκB degradation and NFκB phosphorylation, was detected by **c** flow cytometry and **d** by Western blot after MDP (10 µg/ml) stimulation of patient and control (*n* = 5) peripheral blood cells. **e** Production of IL-1β, TNFα, IL-6 and IL-12p70 after MDP (10 µg/ml) or LPS (1 µg/ml) stimulation of patient and control (*n* = 5) PBMCs was detected by Luminex.

Moreover, XIAP is involved in NOD2 signalling; hence, we investigated whether the pathway was affected. Stimulation of NOD2 with muramyl dipeptide (MDP) leads to activation of NFκB and MAPK. We focused on the phosphorylation of the MAP kinases p38 and Erk (Fig. 4a), and observed diminished levels of kinase phosphorylation in response to MDP in patient monocytes detected by flow cytometry. Western blot analysis of MAPK activation confirmed this assessment (Fig. 4b). Furthermore, we examined the NFκB pathway activation after MDP stimulation, expressed as IκB (inhibitor of κB) degradation, and NFκB phosphorylation by flow cytometry and Western blot. Degradation of IκB leads to NFκB activation and its translocation to the nucleus. As anticipated, we detected neither inhibited IκB degradation in the patient’s samples (Fig. 4c, d) nor NFκB phosphorylation in response to MDP stimulation. However, the patient’s cells were able to phosphorylate MAPKs, as well as activate the NFκB pathway in response to PMA or TNFα stimulation (Supplementary Fig. 3A), suggesting that only the NOD2 pathway was affected. Next, we assessed cytokine production (IL-1β, IL-6 and TNFα) after stimulation of patient PBMCs with MDP and lipopolysaccharide (LPS) (Fig. 4e) using the Luminex method. The patient’s cells produced decreased levels of cytokines after MDP stimulation compared with the healthy controls; however, in response to LPS stimulation, the patient’s PBMCs produced comparable levels of cytokines, confirming defective NOD2 signalling in the patient’s cells.

### XIAP deficiency affects T-cell homoeostasis

**Fig. 5:**
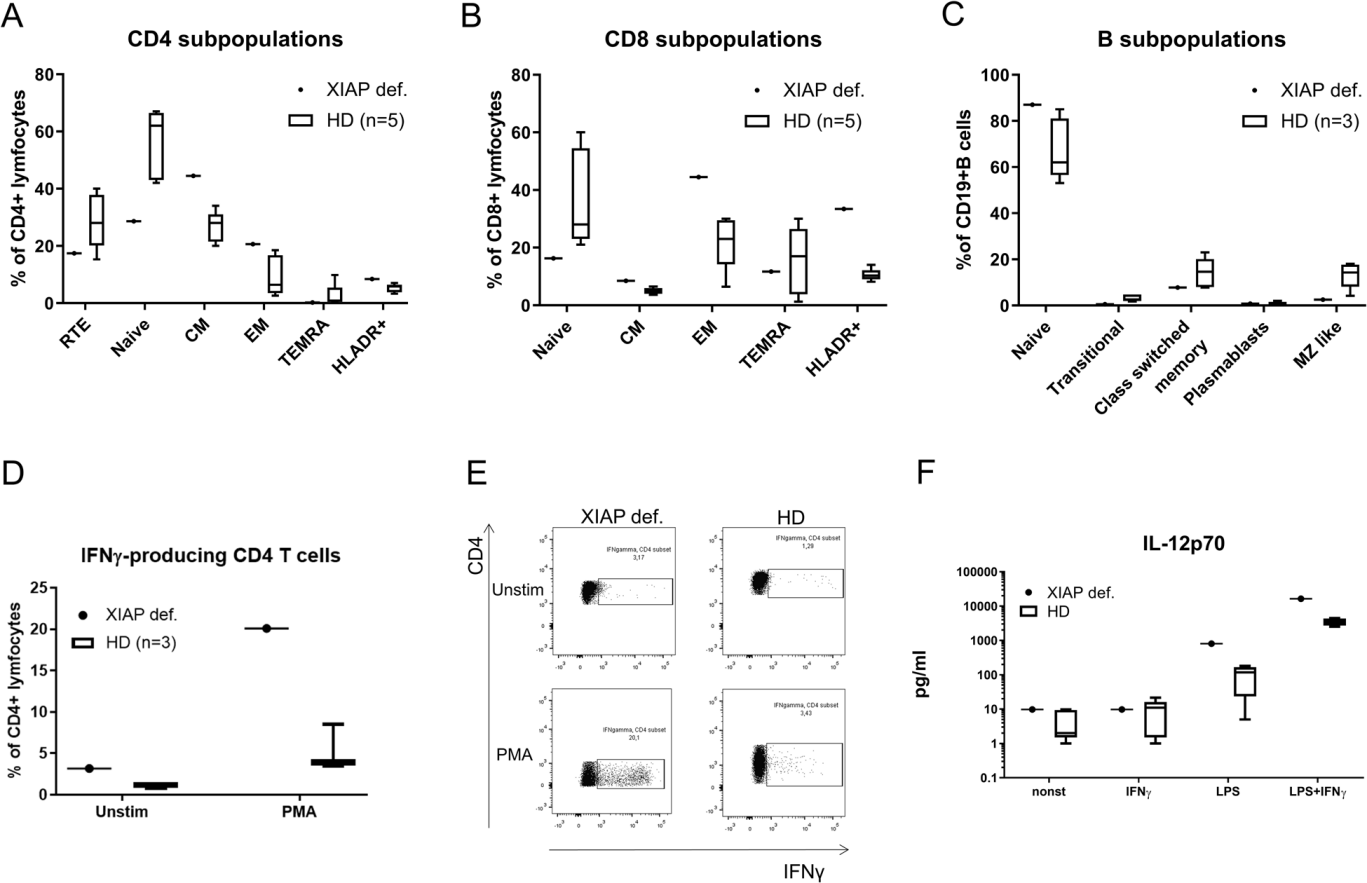
T cells. Proportion of **a** CD4+, **b** CD8+ T cells and **c** B-cell subpopulations of the patient and control (*n* = 3) samples detected by flow cytometry. **d**, **e** Percentage of IFNγ-producing T cells upon PMA (20 ng/ml) stimulation for 6 h. **e** IL-12p70 production upon stimulation with LPS (1 µg/ml), IFNγ (1 µg/ml) or their combination of patient and control (*n* = 5). PBMCs were detected by Luminex.

To test whether XIAP deficiency and impaired apoptosis influenced the distribution of the patient’s B- and T-cell subpopulations, we analyzed these subsets. The gating strategies used to distinguish between naive, central memory (CM), effector memory (EM), terminal effector T cells re-expressing CD45RA (TEMRA), recent thymic emigrants (RTEs) and B-cell subsets, are illustrated in Supplementary Fig. 4. The analysis showed a shift towards mature stages of CD4+ and CD8+ T cells in the patient samples (Fig. 5a, b). We found a noteworthy increase in the count of CM and EM, and a reduction in naive forms of the T cells; however, the percentage of RTEs was unaffected. Consequently, we analyzed the patient’s T-lymphocyte ability to produce IFNγ by flow cytometry. The patient’s T cells produced higher levels of IFNγ even in the unstimulated state, which was significantly elevated upon PMA stimulation. The percentage of IFNγ-producing CD4+ T cells (20.1%) was considerably higher than that of the healthy donors (5.3%) (Fig. 5d, e). In addition, analysis of activation marker expression on T cells, HLA-DR as a marker of chronic activation, and CD69 as the earliest activation marker, revealed a shift towards late stages of activation. HLA-DR expression was threefold higher on the CD8+ T cells and twofold higher on CD4+ T cells than it was in the healthy controls (Fig. 5a, b). CD69 expression was unaffected (Supplementary Fig. 3C). T-cell proliferation was negligibly decreased (55.2% patients; controls 74.7% after PMA and ionomycin stimulation) (Supplementary Fig. 3D). Moreover, we also observed higher production of IL-12 in response to LPS in patient PBMCs, supporting a Th1-polarizing environment (Fig. 5f). No significant differences were found in the B-cell department (Fig. 5c).

## Discussion

Here, we report the case of a patient who developed adult-onset IBD refractory to treatment and complicated by several episodes of HLH, and for whom WES revealed a novel previously unpublished c.266delA mutation in the *XIAP* (*BIRC4*) gene that led to its loss of function. HLH and IBD are the most common first manifestations of XIAP deficiency, which usually occurs in the first few years of life, and for which the potentially lethal outcome requires HSCT^13^. Adult-onset HLH and IBD associated with XIAP deficiency, although rare, have also been described^17,22^. In a large cohort of 54 XIAP-deficient patients, IBD manifestation was the main clinical feature in 17 of them. The remaining patients usually manifested with HLH as a major disease complication. The average age at the time of diagnosis of IBD was 11 years (range 3 months–41 years) compared with patients manifested with HLH (average age 6.5 years and range 0.1–23 years). In our patient, IBD manifested at the age of 17 and HLH at the age of 20. The majority of the first HLH attacks was associated with EBV infection; however, HHV6 and HSV1 were identified as potential triggers as well. IBD-related complications were the main cause of death in three of them at the average age 24 years (range 4–42 years) and after 4 years of disease duration (range 0–7 years). Interestingly, only four patients presented in a form of the adult-onset IBD^22,23^.

Most of the *XIAP* mutations identified in XLP-2 patients are nonsense mutations, frameshift mutations or deletions that cause severe aberrations in the encoded protein or loss of its expression. They are distributed along all coding exons^10,13,24–26^. Neither type nor position of the mutation, as well as residual protein expression, do not correlate with the clinical manifestation and severity of the disease^23^.

We report a novel deletion mutation c.266delA, resulting in a premature stop codon (p. Asn89fs*41), loss of protein expression and, as a consequence, a patient suffering from XLP-2 and lower expression in his mother, who is a healthy carrier of the mutation.

XIAP-deficient T cells are characterized by a high susceptibility to apoptosis ex vivo in response to apoptotic stimulus or upon activation^17,23,27^. Indeed, we observed an enhanced level of apoptosis in response to staurosporine, an inducer of apoptosis, as well as upon activation by PMA. Interestingly, we also observed increased spontaneous apoptosis in patient lymphocytes, which was reduced when a caspase inhibitor was applied. However, the sensitivity to apoptosis of T cells was found to have no influence on circulating blood lymphocyte numbers in patients^27^. Accordingly, circulating T-cell numbers were in the normal range in the patient, although we observed a shift to their more mature stages. Considering T-lymphocyte function, the expansion and proliferation of virus-specific T lymphocytes might be compromised in XIAP deficiency. XIAP-deficient patients suffer from an increased risk of EBV infections, and in a mouse model^28^, XIAP and cIAP1 were required for the survival and expansion of virus-specific T cells. In addition, defective NOD2 signalling might also contribute to a higher risk of EBV infection^29,30^. Apoptosis may be further ameliorated by increased production of IFNγ, which further enhances the expression of pro-apoptotic genes (such as *BAX*, *BAK1* and/or *XAF1*)^31^.

The aforementioned shift in the spectrum of T lymphocytes to their more mature stages seems to be related to the alteration of the apoptosis process. It has been previously reported that T lymphocytes at different stages of development have different sensitivities to apoptosis, possibly resulting from different expression of pro- and anti-apoptotic proteins^32,33^. These differences may lead to a significant reduction in naive and the subsequent survival of the mature memory forms of T cells, including CM and EM T cells, as observed in the patient.

HLH is the most severe and life-threatening manifestation in patients with XIAP deficiency, but the exact mechanism by which mutated *XIAP* results in HLH manifestations is not entirely clear. The mechanism differs from other genetic disorders associated with HLH, such as XLP-1, in which the impaired cytotoxic responses by CD8+ lymphocytes and NK cells result in exaggerated amounts of IFNγ and the activation of macrophages, thus explaining the positive effect of the IFNγ blockade on the outcome of HLH^15^. The patient’s T lymphocytes produced markedly higher levels of IFNγ in comparison with the healthy donors, even though XIAP deficiency was not connected with defects in the cytotoxic responses by CD8+ lymphocytes or NK cells, as is typical in XLP-1^27^. The shift towards the Th1 immune response and increased production of IFNγ was further supported by the overproduction of IL-12, a crucial cytokine for Th1 polarization^34^. Observations in a mouse model propose, as a possible explanation, that HLH is due to NLRP3 inflammasome dysregulation and increased proinflammatory cytokine production^35,36^. Although it is still unclear whether XIAP in humans also acts as an NLRP3 inhibitor, impairment to this control might represent a key pathological mechanism. XIAP-deficient mice also develop splenomegaly when treated with an activator of the NLRP3 inflammasome^36^; therefore, this mechanism may explain two of three typical pathologies associated with XIAP deficiency. Interestingly, mutations in the human NLRC4 inflammasome were identified in patients suffering from recurrent HLH and autoinflammation, supporting a role of the inflammasome in HLH^37,38^. However, we observed only slightly higher IL-1β and TNFα production in the patient in response to LPS stimulation.

In line with previous reports, the patient displayed diminished proinflammatory cytokine production after NOD2 ligand stimulation^12,39,40^, thus connecting the potential role for altered NOD2 signalling with IBD in XIAP patients. NOD2 mutations represent a strong genetic risk factor for CD^11^, as NOD2-impaired secretion of cytokines and an altered gut microbiome may disturb intestinal homoeostasis. Like HLH, IFNγ is one of the most important cytokines in CD pathophysiology. Indeed, as shown here for the XIAP-deficient patient, altered NOD2-mediated signalling and high IFNγ production by T cells might explain, in an analogy to CD, the gastrointestinal IBD-like presentation as a feature of XIAP deficiency.

Taken together, our data reveal a novel mutation in a patient suffering from recurrent HLH, IBD and splenomegaly, typical conditions associated with XIAP deficiency. The deletion mutation leads to loss of XIAP expression, and it functions as a negative regulator of apoptosis. The absence of XIAP clearly leads to enhanced cell death, which may amplify inflammation. XIAP deficiency negatively influences MDP-induced NOD2 signalling, with implications for IBD. Changes in innate immunity, highlighted together with the role of IFNγ, contribute to XLP-2 pathogenesis and complex clinical presentation. Whereas HSCT in patients with the early onset of the disease represents a method of choice, in adult patients, such as in the index patient in our study, the therapeutic options are more limited. Emapalumab, a monoclonal antibody that targets IFNγ, was approved for the treatment of relapsed/refractory HLH^14^ with a possible influence on the symptoms of CD, and anti-IL-12/23 (ustekinumab) therapy^41^ is also available. The overlap in pathogenetic mechanisms gives hope for the use of this strategy to treat XIAP deficiency.

## Patient and methods

Informed written consent was obtained from all subjects involved in the study and all controls in accordance with the Declaration of Helsinki, and according to the procedures established by the Ethical Committee of our institution.

### Whole-exome sequencing

WES was performed on a NextSeq 500 instrument (Illumina, San Diego, CA), and sequencing libraries were prepared using the SureSelectXT Human All Exon V6 + UTR kit (Agilent Technologies, Santa Clara, CA). Sequencing reads were aligned against the human reference genome hg19 by BWA^42^, and variant calling was performed using SAMtools^43^ and VarScan 2^44^ and their annotation using SnpEff^45^.

### Apoptosis

Peripheral blood was collected from the patient and healthy volunteers into EDTA-coated tubes. Peripheral blood mononuclear cells (PBMCs) were isolated using Ficoll-Paque (GE Healthcare Biosciences, Uppsala, Sweden). The obtained cells were resuspended in RPMI 1640 medium with a sodium bicarbonate buffer system supplemented with 2% autologous serum, 1% penicillin and streptomycin and 1% GlutaMAX (Thermo Fisher Scientific, Waltham, CA, USA). PBMCs (10^6^/ml) stimulated with staurosporine (1 mmol) (Abcam, Cambridge, UK) for 4 and 6 h, PMA (50 ng/ml) (Sigma-Aldrich, Darmstadt, Germany) for 4 h or left untreated for 4, 6, 24 and 48 h. When indicated, 20 µM Z-VAD-FMK was added in the culture 30 min before apoptosis induction. Then, the cells were washed in Annexin V binding buffer and stained with Annexin V–Dyomics 647 (EXBIO) and DAPI (Thermo Fisher Scientific).

### FLICA staining

Active caspase-3 and -7 were detected using a FLICA caspase-3 and 7 assay kit (Thermo Fisher Scientific). PBMCs were stimulated as described above prior to treatment with the fluorescein-labelled inhibitor Z-YVAD-fmk (10 µM) for 1 h at 37 °C and CD3-A700 (clone MEM-57) (EXBIO, Prague, Czech Republic). The cells were washed three times and analyzed by flow cytometry with a FACS Fortessa flow cytometer (BD Biosciences, San Diego, CA, USA).

### Phospho-flow cytometry

Detection of MAPK and NFκB activation was performed according to a previously published protocol^46^. Briefly, peripheral blood was stimulated with 10 μg/ml MDP (InvivoGen, San Diego, CA, USA) for 20 min at 37 °C or left unstimulated. Subsequently, the cells were fixed using 4% formaldehyde for 10 min at 25 °C, erythrocytes were lysed using 0.1% Triton X-100 (Sigma-Aldrich) for 15 min at 37 °C and the leukocytes were permeabilized using 80% ice-cold methanol for 30 min.

The following antibodies were used: CD3—A700 (clone MEM-57), CD14—PEDy594 (EXBIO) and CD19—PC7 (clone J3-119) (Beckman Coulter, USA, Brea, USA), phospho38 (Thr180)—A647 (#4552 S), phosphoErk1/2 (Thr202/Tyr204)—A488 (#4374 S), phosphoSAPJ/JNK (Thr183/185)—PE (#5755 S) (Cell Signaling, Denvers, MA, USA), phosphoNFκB—A647 (#4887) and anti-IκB—A488 (#5743) (both from Cell Signaling).

### Cytokine production

Cytokines were detected using a multiplex Luminex cytokine-fluorescent bead-based immunoassay (Merck Millipore, Beerlengton, MA, USA) with cell-free supernatants. A total of 2 × 10^5^ PBMCs were stimulated with MDP (10 μg/ml) (InvivoGen), *E. coli* LPS (1 μg/ml) (Sigma-Aldrich) or left untreated for 24 h.

### T- and B-cell analysis

Immunophenotyping of T and B cells was performed according to a previously published protocol^47^, and the gating strategy is shown in Supplementary Fig. 2B.

For IFNγ-producing cell detection, we applied an already-published protocol^46^.

### T-cell proliferation

The proliferation of CD3+ T lymphocytes was determined according to a previously published protocol^48^.

### RT-PCR

PBMCs were stimulated as stated in the ‘Apoptosis’ section. RNA isolation, reverse transcription and RT-PCR were performed according to a previously published protocol^49^. TaqMan primer/probe sets (Thermo Fisher Scientific) were used. The sample data were matched to a standard curve generated by amplifying serially diluted products using the same PCR, and normalized to *GAPDH* (TIB Molbiol, Berlin, Germany) to obtain the relative expression value. Real-time assays were run on an FX96 cycler (Bio-Rad). The primer/probe sets are available from the authors upon request.

### Western blotting

Detection of proteins was performed according to a previously published protocol^46^. The membranes were incubated with the following primary antibodies: anti-XIAP (clone D2Z8W), anti-β-actin (clone D6A8), anti-GAPDH (clone D16H11), IκB anti (clone L35A5), anti-NFκB (clone D14E12) (all from Cell Signaling), anti-tubulin (clone TU-07), anti-HSP-90 (clone MBH90AB) (both from Exbio), anti-Erk1/2 (ab17942), anti-p-Erk1/2 (ab76299), anti-p-p38 (ab4822), anti-p38 (ab170099), anti-p-JNK1/2/3 (ab124956), anti-JNK1/2/3 (ab208035) and anti-p-NFκB (ab76302) (all from Abcam) overnight, followed by incubation with peroxidase-conjugated anti-rabbit or anti-mouse secondary antibodies for 2 h. The membranes were developed using SuperSignal West Femto (Thermo Fisher Scientific).

## Supplementary information

Supplementary Figure Legends

Supplementary Figure 1

Supplementary Figure 2

Supplementary Figure 3

Supplementary Figure 4

Supplementary Table

Supplementary Material
