# Supplementary material for: Novel XIAP mutation causing enhanced spontaneous apoptosis and disturbed NOD2 signalling in a patient with atypical adult-onset Crohn’s disease
Source: Cell Death Dis. 2020 Jun 8;11(6):430. doi: 10.1038/s41419-020-2652-4 (PMC7280281; doi:10.1038/s41419-020-2652-4)
Supplement: Supplementary file 1 — Supplementary Figure Legends [file 41419_2020_2652_MOESM1_ESM.docx]

**Supplementary Table 1:** Laboratory values during HLH episode and remission, including complete blood count and differential lymphocyte subpopulations (NK – natural killer), biochemistry (ALT – alanine aminotransferase, AST – aspartate aminotransferase, GGT- gamma-glutamyl transferase, ALP – alkaline phosphatase, bili – bilirubin, TAG – triacylglycerol, and Chol – cholesterol) and inflammatory markers (C reactive protein), 🡹🡻 - value above and below the reference range, respectively.

**Supplementary Figure 1:** **A.** Patient’s clinical manifestations and therapy. Uncropped membranes from the Western blot analyses of **B.** XIAP, **C.** β actin **D.** and GAPDH. Expression of housekeeping proteins **E.** tubulin and HSP90 in patient and control PBMCs.

**Supplementary Figure 2: Apoptosis.** Patient PBMCs were treated with staurosporine (1 mmol) and 50 ng/ml PMA for 2, 4 and 6 hours or left untreated for an additional 24 and 48 hours. When indicated the cells were pre-treated with 20µM Z-VAD-fmk for 30 minutes. The level of spontaneous and induced apoptosis was detected by **A**. FLICA, in which the fluorescein-labelled inhibitor Z-YVAD-fmk is bound to activated caspase -3 and 7, signal as detected by flow cytometry. **B.** The level of spontaneous apoptosis detected by flow cytometry of cells stained with Annexin V and DAPI. Annexin+ DAPI cells were considered to be undergoing early apoptosis.

**Supplementary Figure 3:** **A.** MAPK and NFκB signalling pathways activation upon PMA or TNFα stimulation for 20 minutes of patient and control peripheral blood. **B.** Uncropped membranes from the Western blot analyses for MAPK and NFκB signalling pathways activation. **C.** CD69 expression on T cells expressed as MFI. **D.** T cell proliferation. Proliferating cells are expressed as Ki67+CD4+ T cells.

**Supplementary Figure 4:** Gating strategies for determining the populations of T and B cells in the patient.
