# Supplementary figures and images for: Novel XIAP mutation causing enhanced spontaneous apoptosis and disturbed NOD2 signalling in a patient with atypical adult-onset Crohn’s disease
Source: Cell Death Dis. 2020 Jun 8;11(6):430. doi: 10.1038/s41419-020-2652-4 (PMC7280281; doi:10.1038/s41419-020-2652-4)

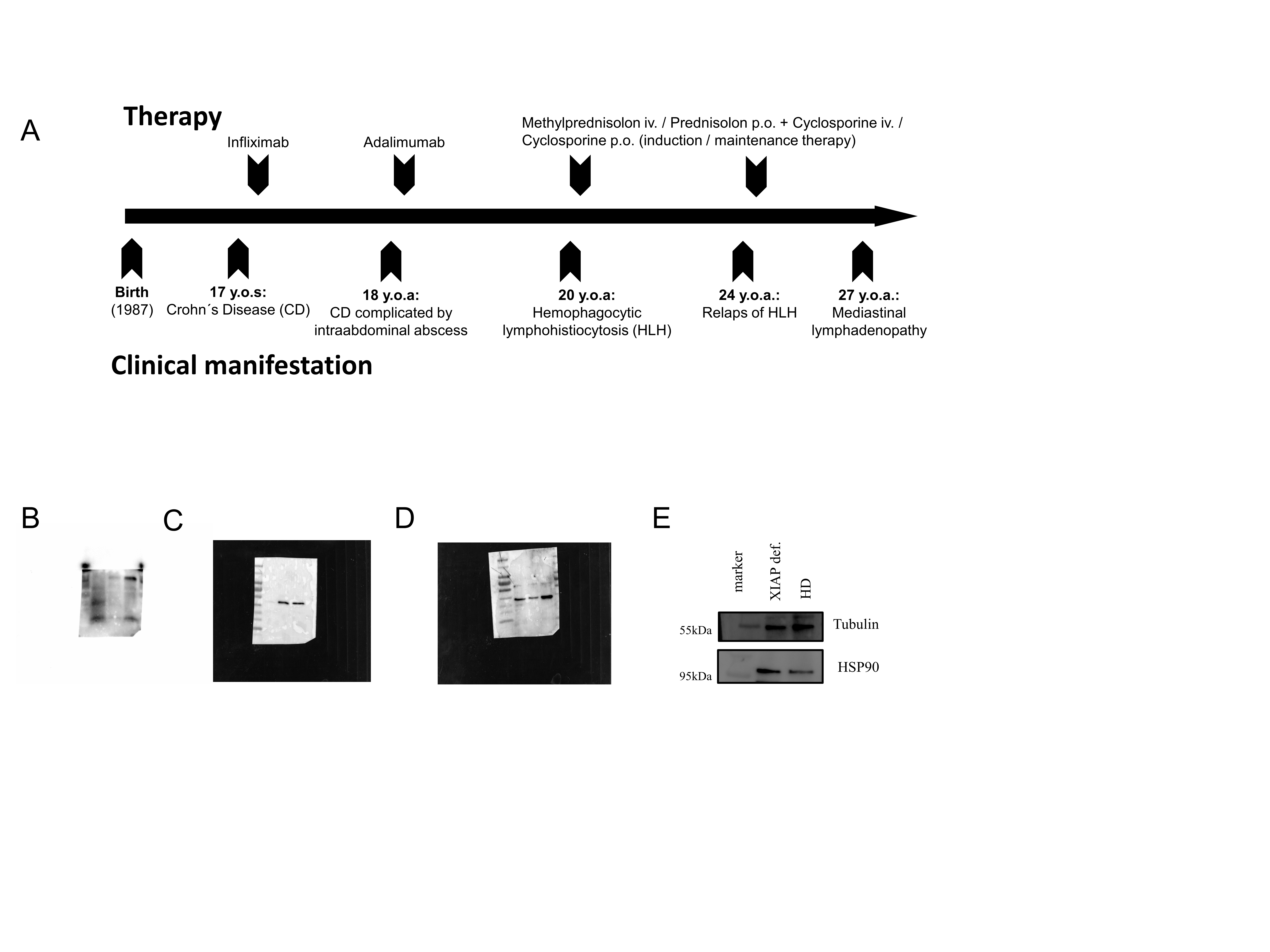

Supplement: Supplementary file 2 — Supplementary Figure 1 [file 41419_2020_2652_MOESM2_ESM.tif]

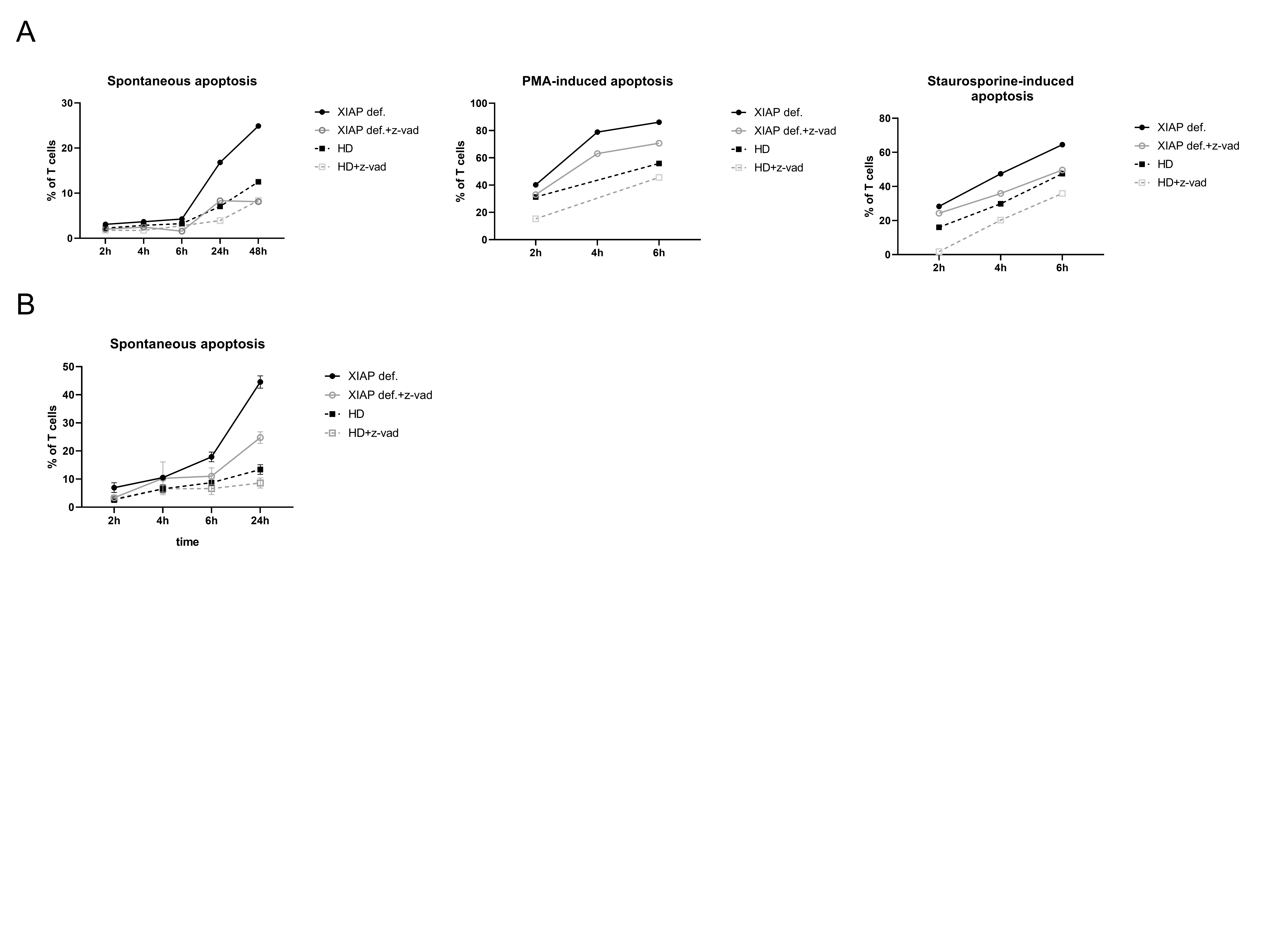

Supplement: Supplementary file 3 — Supplementary Figure 2 [file 41419_2020_2652_MOESM3_ESM.tif]

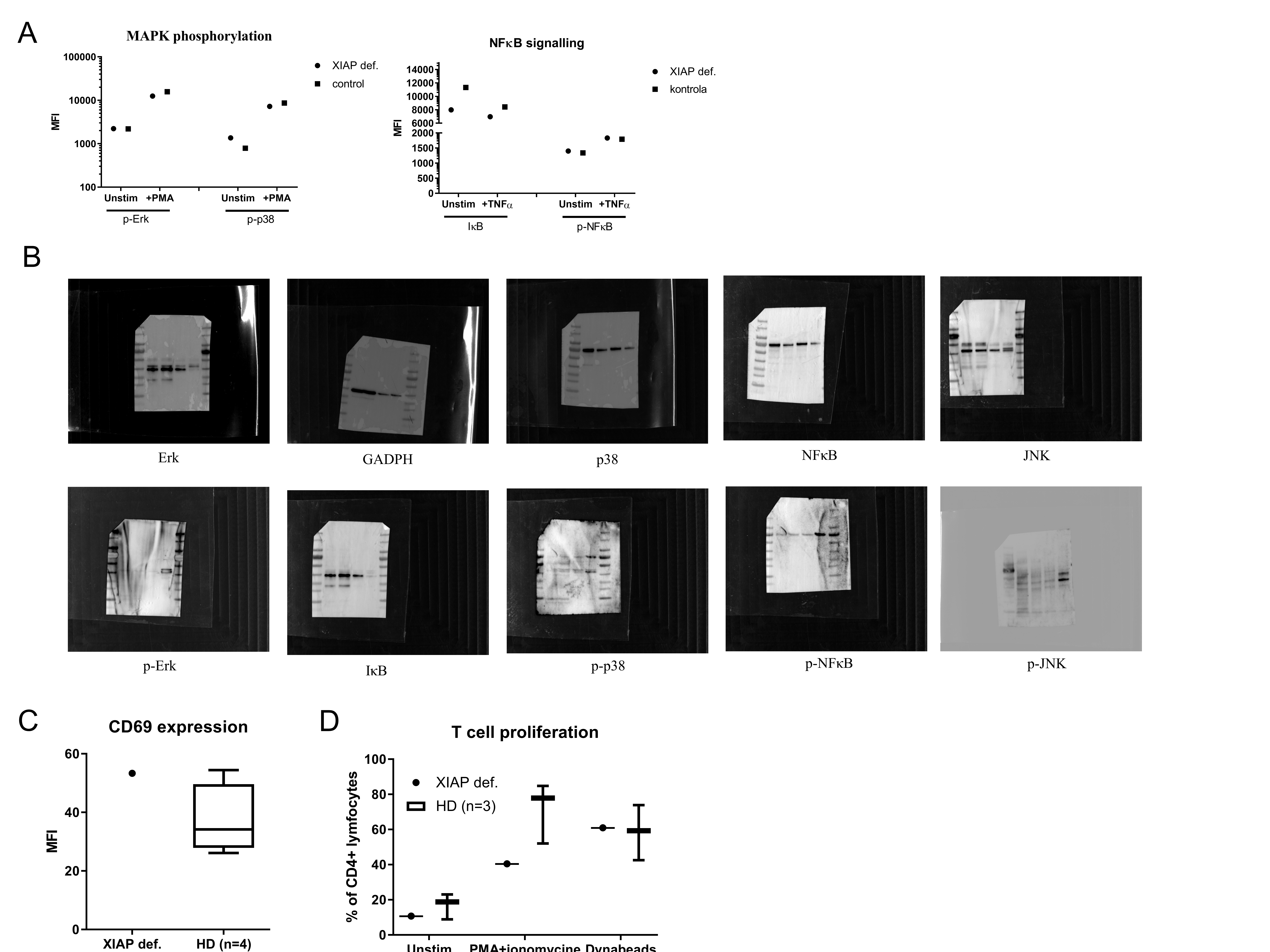

Supplement: Supplementary file 4 — Supplementary Figure 3 [file 41419_2020_2652_MOESM4_ESM.tif]

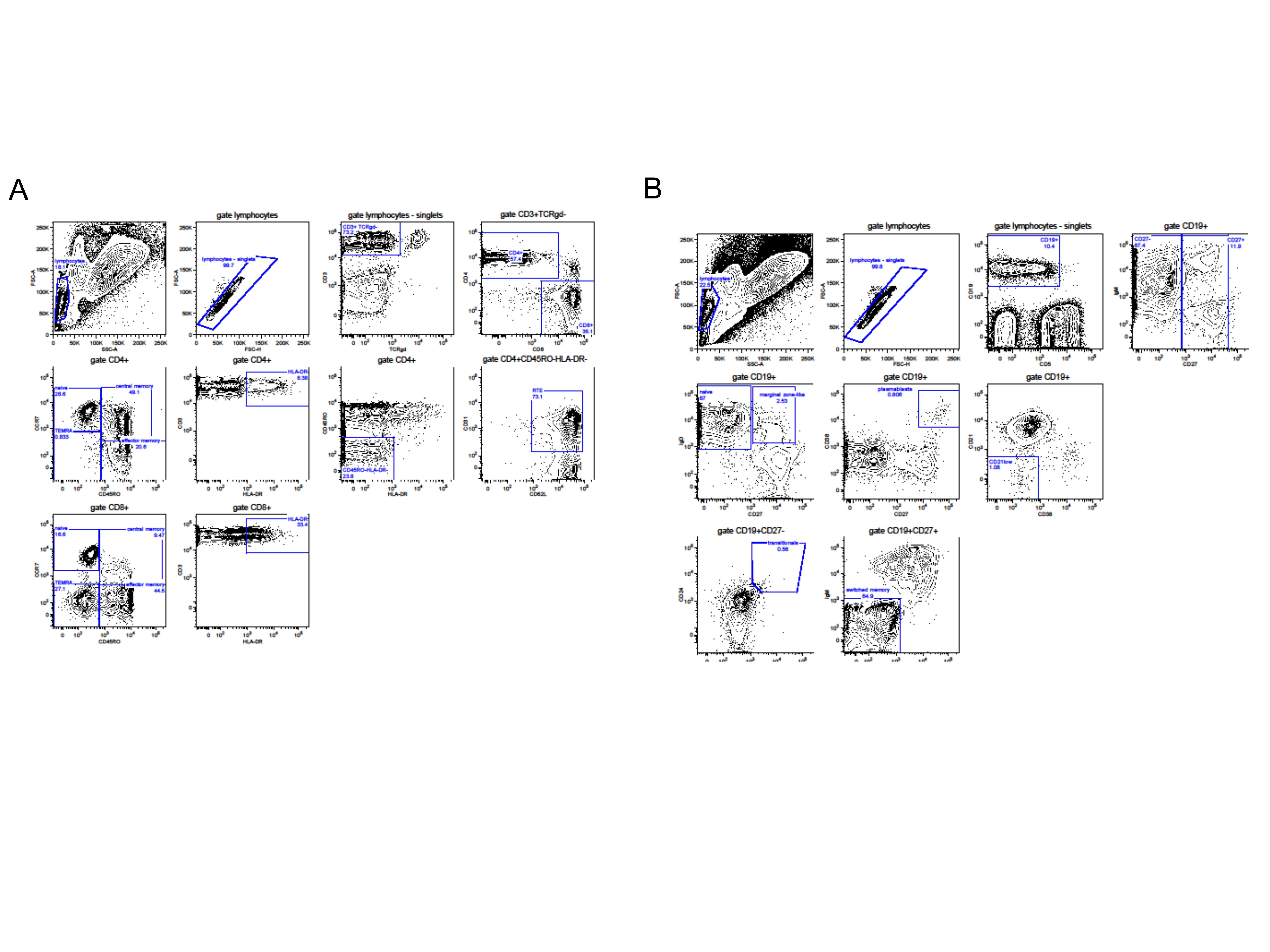

Supplement: Supplementary file 5 — Supplementary Figure 4 [file 41419_2020_2652_MOESM5_ESM.tif]
