# Supplementary material for: Novel XIAP mutation causing enhanced spontaneous apoptosis and disturbed NOD2 signalling in a patient with atypical adult-onset Crohn’s disease
Source: Cell Death Dis. 2020 Jun 8;11(6):430. doi: 10.1038/s41419-020-2652-4 (PMC7280281; doi:10.1038/s41419-020-2652-4)

NOVEL *XIAP* MUTATION CAUSING ENHANCED SPONTANEOUS APOPTOSIS AND DISTURBED NOD2 SIGNALLING IN A PATIENT WITH ATYPICAL ADULT ONSET CROHN'S DISEASE

Short running title: A novel XIAP mutation

Zuzana Parackova[1], Tomas Milota[1], Petra Vrabcova[1], Jitka Smetanová[1], Michael Svaton[2], Tomas Freiberger[3, 4], Veronika Kanderova[2], Anna Sediva[1]

[1] Department of Immunology, 2nd Faculty of Medicine Charles University, University Hospital in Motol, V Uvalu 84, Prague, Czech Republic
[2] CLIP - Childhood Leukaemia Investigation Prague, Department of Paediatric Haematology and Oncology, 2nd Faculty of Medicine, Charles University and University Hospital Motol, Prague, Czech Republic
[3] Molecular Genetics Laboratory, Center of Cardiovascular Surgery and Transplantation, Brno, Czech Republic
[4] Faculty of Medicine, Masaryk University, Brno, Czech Republic

Correspondence:

Zuzana Paračková, Department of Immunology, 2nd Faculty of Medicine Charles University, Faculty Hospital in Motol, V Uvalu 84, Prague 5, 15006, +420224435971, zuzana.parackova@fnmotol.cz

Keywords: XIAP, BIRC4, XLP, NOD2, apoptosis, HLH, IFNγ, Crohn's disease

Supplementary Table 1: Laboratory values during HLH episode and remission, including complete blood count and differential lymphocyte subpopulations (NK – natural killer), biochemistry (ALT – alanine aminotransferase, AST – aspartate aminotransferase, GGT- gamma-glutamyl transferase, ALP – alkaline phosphatase, bili – bilirubin, TAG – triacylglycerol, and Chol – cholesterol) and inflammatory markers (C reactive protein), 🡅🡇 - value above and below the reference range, respectively.

**Hematology**	**HLH**	**Remission**	**Reference value**
Leukocytes (E9/l))	↓ **2.8**	5.3	4-10
Neutrophils (%)	↑ **77.5**	58.8	45-70
Monocytes (%)	↓ **15.5**	↓ **9.9**	20-45
Bazophiles (%)	↑ **5.6**	0.2	0-2
Eozinophiles(%)	0	1.1	0-5
HGB (g/l)	↓ **0**	147	135-175
PLT (E9/l)	↓ **75**	208	150-400
Lymphocytes (%)	↑ **67**	29.8	20-45
CD3 (%)	83	86	57-94
CD4 (%)	40	47	20-70
CD8 (%)	37	33	10-48
CD19 (%)	12	9	4-23
NK (%)	↓ **3**	↓ **4**	6-33
CD3 HLADR (%)	↑**17**	15	0-15
**Biochemistry**	**HLH**	**Remission**	**Reference value**
ALT (ukat/l)	0.4	0.45	0.17-0.78
AST (ukat/l)	0.18	0.32	0.16-0.72
GGT (ukat/l)	↑ **9.01**	0.66	0.14-0.84
ALP (ukat/l)	↓ **0.4**	1.38	0.66-2.20
Bili (umol/l)	↑ **24**	13.5	5-21
TAG (mmol/l)	↑ **4.65**	↑ **2**	0.7-1.7
Chol (mmol/l)	4.4	↑ **5.5**	3.4-5.0
Fe (umol/l)	↓ **1.9**	11.1	7.2-29
Ferritin (ug/l)	↑ **16,500**	62	22-322
CRP (mg/l)	↑ **14**	↑ **55.3**	0-5

Supplementary Figure 1: A. Patient's clinical manifestations and therapy. Uncropped membranes from the Western blot analyses of **B.** XIAP, **C.** β actin **D.** and GAPDH. Expression of housekeeping proteins **E.** tubulin and HSP90 in patient and control PBMCs.

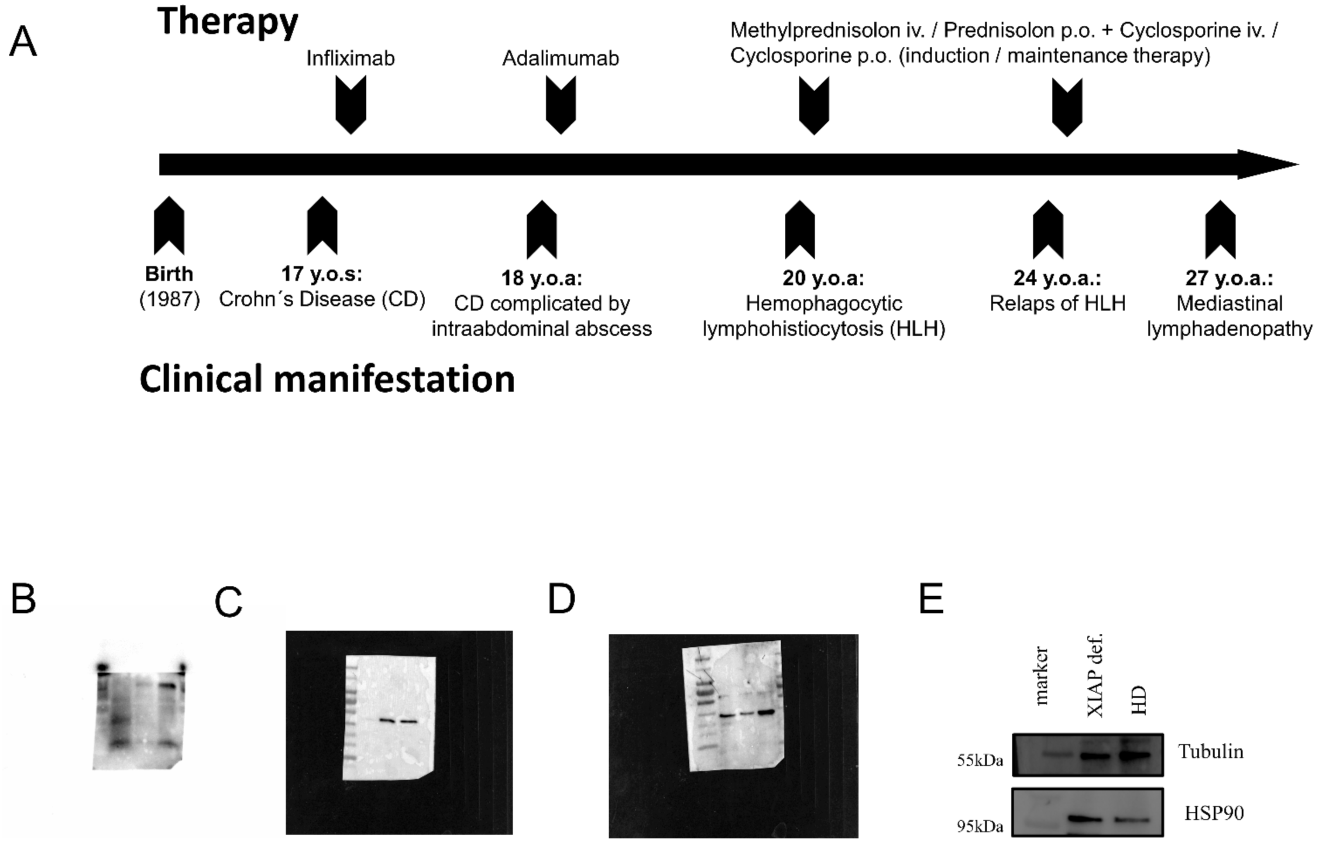

Supplementary Figure 2: Apoptosis. Patient PBMCs were treated with staurosporine (1 mmol) and 50 ng/ml PMA for 2, 4 and 6 hours or left untreated for an additional 24 and 48 hours. When indicated the cells were pre-treated with 20µM Z-VAD-fmk for 30 minutes. The level of spontaneous and induced apoptosis was detected by **A**. FLICA, in which the fluorescein-labelled inhibitor Z-YVAD-fmk is bound to activated caspase -3 and 7, signal as detected by flow cytometry. **B.** The level of spontaneous apoptosis detected by flow cytometry of cells stained with Annexin V and DAPI. Annexin+ DAPI cells were considered to be undergoing early apoptosis.

A

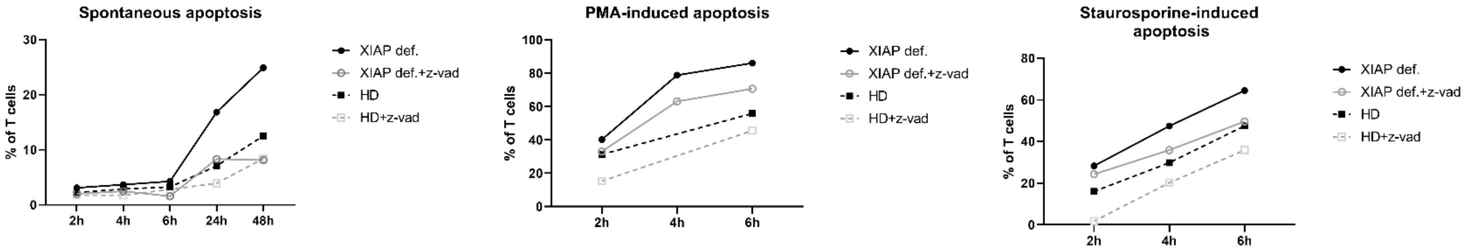

B

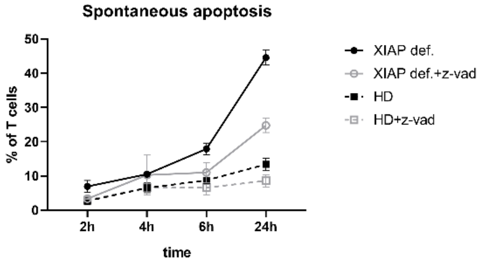

Supplementary Figure 3: A. MAPK and NFκB signalling pathways activation upon PMA or TNFα stimulation for 20 minutes of patient and control peripheral blood. **B.** Uncropped membranes from the Western blot analyses for MAPK and NFκB signalling pathways activation. **C.** CD69 expression on T cells expressed as MFI. **D.** T cell proliferation. Proliferating cells are expressed as Ki67+CD4+ T cells.

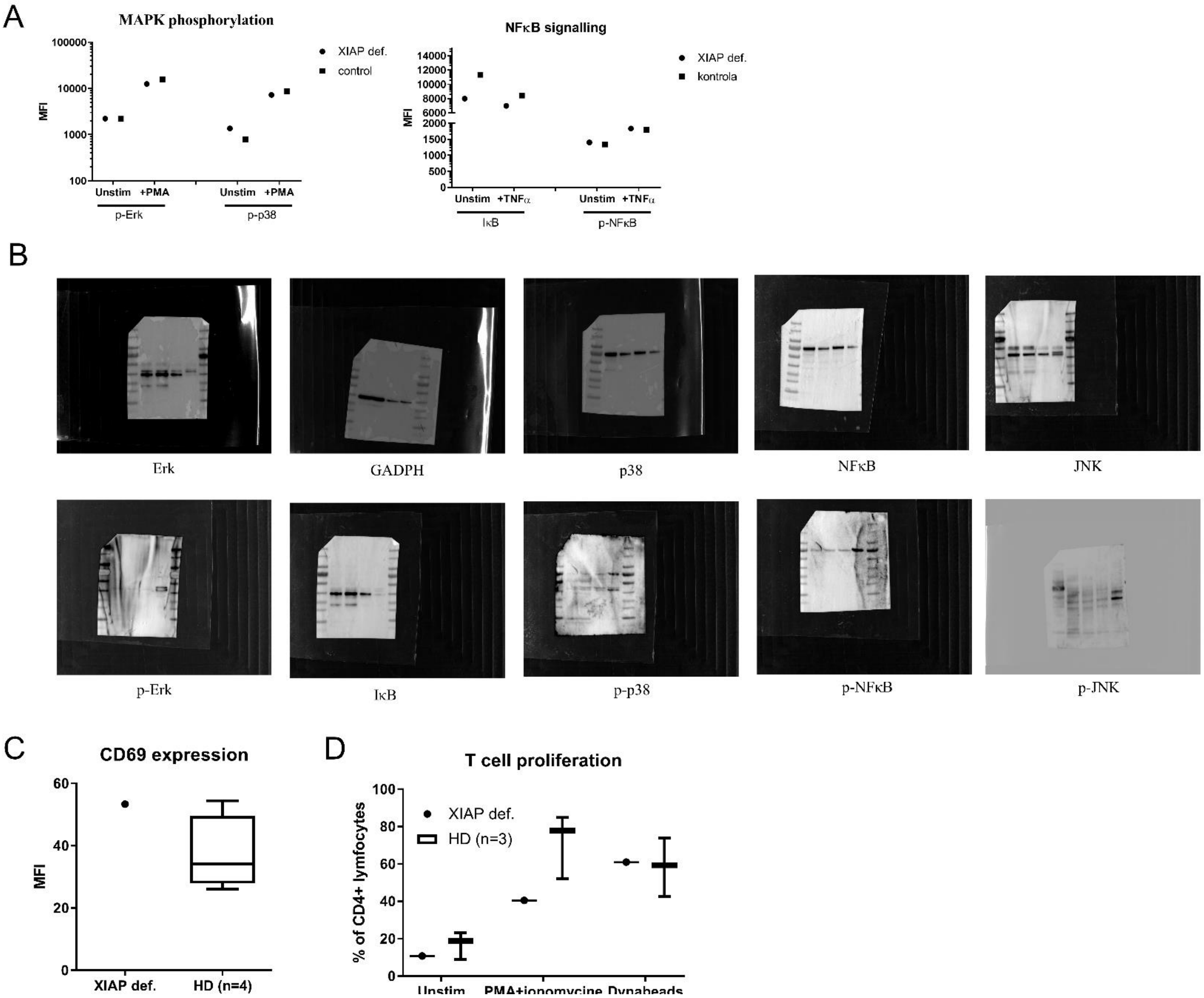

Supplementary Figure 4: Gating strategies for determining the populations of T and B cells in the patient.

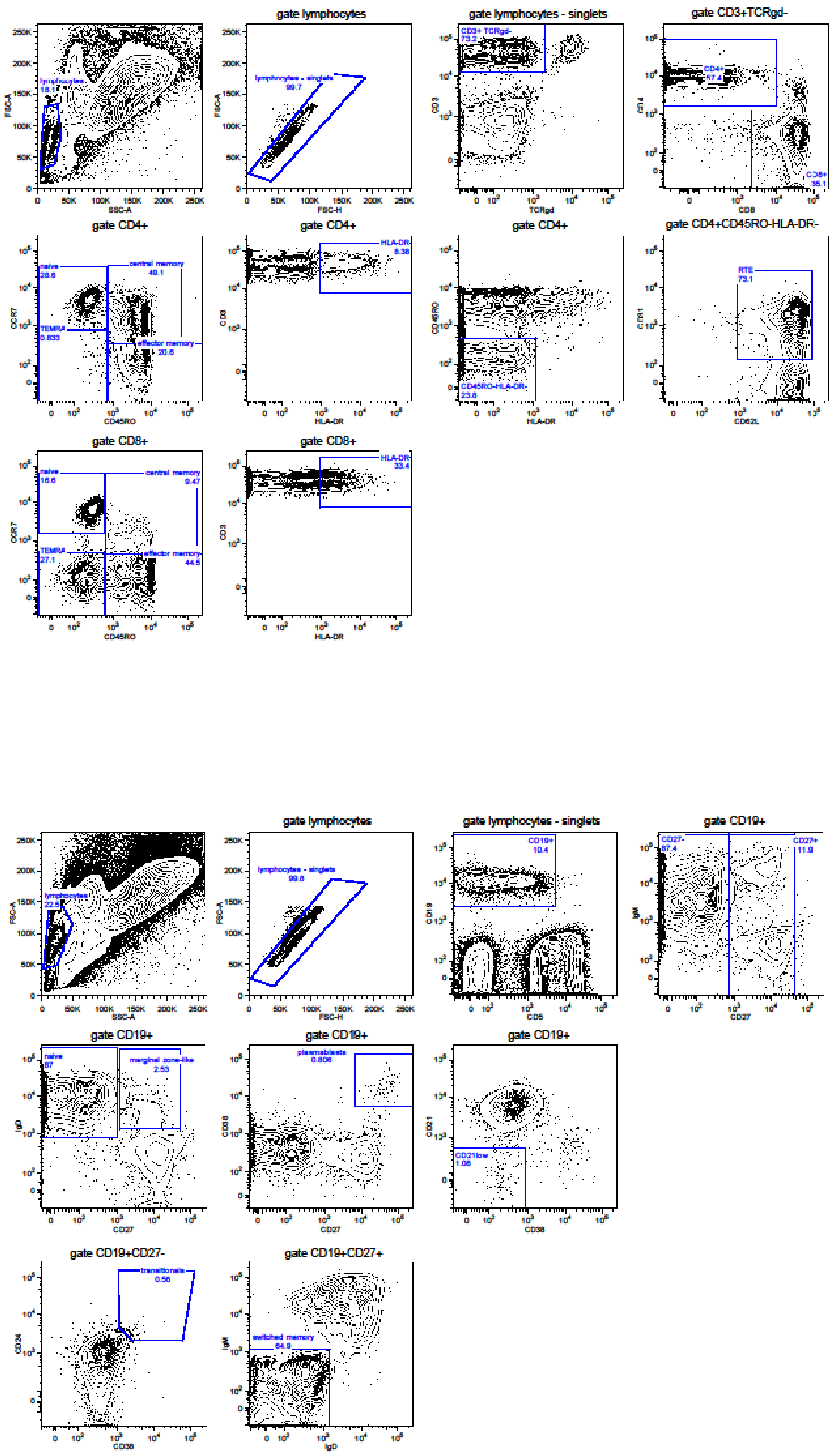

Supplement: Supplementary file 7 — Supplementary Material [file 41419_2020_2652_MOESM7_ESM.pdf]
